# *Mycobacterium tuberculosis* expressing phospholipase C subverts PGE_2_ synthesis and induces necrosis in alveolar macrophages

**DOI:** 10.1186/1471-2180-14-128

**Published:** 2014-05-19

**Authors:** Patricia A Assis, Milena S Espíndola, Francisco WG Paula-Silva, Wendy M Rios, Priscilla AT Pereira, Sylvia C Leão, Célio L Silva, Lúcia H Faccioli

**Affiliations:** 1 Departamento de Análises Clínicas, Toxicológicas e Bromatológicas, Faculdade de Ciências Farmacêuticas de Ribeirão Preto, Universidade de São Paulo, Av Cafe, s/n, Ribeirão Preto, SP 14040-903, Brazil; 2 Departamento de Microbiologia, Imunologia e Parasitologia, Escola Paulista de Medicina, Universidade Federal de São Paulo, São Paulo, Brazil; 3 Departamento de Bioquímica e Imunologia, Faculdade de Medicina de Ribeirão Preto, Universidade de São Paulo, Ribeirão Preto, Brazil

**Keywords:** Mycobacterium, Lipid mediator, Phospholipase C, Cell death, Macrophage necrosis, Prostaglandins

## Abstract

**Background:**

Phospholipases C (PLCs) are virulence factors found in several bacteria. In *Mycobacterium tuberculosis* (Mtb) they exhibit cytotoxic effects on macrophages, but the mechanisms involved in PLC-induced cell death are not fully understood. It has been reported that induction of cell necrosis by virulent Mtb is coordinated by subversion of PGE_2_, an essential factor in cell membrane protection.

**Results:**

Using two Mtb clinical isolates carrying genetic variations in PLC genes, we show that the isolate 97-1505, which bears *plcA* and *plcB* genes, is more resistant to alveolar macrophage microbicidal activity than the isolate 97-1200, which has all PLC genes deleted. The isolate 97-1505 also induced higher rates of alveolar macrophage necrosis, and likewise inhibited COX-2 expression and PGE_2_ production. To address the direct effect of mycobacterial PLC on cell necrosis and PGE_2_ inhibition, both isolates were treated with PLC inhibitors prior to macrophage infection. Interestingly, inhibition of PLCs affected the ability of the isolate 97-1505 to induce necrosis, leading to cell death rates similar to those induced by the isolate 97-1200. Finally, PGE_2_ production by Mtb 97-1505-infected macrophages was restored to levels similar to those produced by 97-1200-infected cells.

**Conclusions:**

*Mycobacterium tuberculosis* bearing PLCs genes induces alveolar macrophage necrosis, which is associated to subversion of PGE_2_ production.

## Background

*Mycobacterium tuberculosis* (Mtb), the causative agent of tuberculosis, carries different virulence factors, which allow proliferation of the pathogen in the host cell, cell-to-cell spread, and evasion of immune response. Among the most known virulence factors, phospholipases C (PLCs) stand out in several intracellular bacteria, including *Clostridium perfringens, Corynebacterium pseudotuberculosis, Pseudomonas aeruginosa, Staphylococcus aureus*, and *Listeria monocytogen*[1-5]. The most virulent PLC characterised to date is the α toxin (CPA) from *Clostridium perfringens* exhibiting lethal, haemolytic, dermonecrotic, vascular permeabilising, and platelet-aggregating properties [2]. Thus, due to their role in the virulence mechanisms of many bacterial pathogens, the relevance of PLCs during mycobacterial infection has been the subject of investigation [6,7].

*Mycobacterium tuberculosis* PLCs are encoded by four different genes [8]. Three of these genes, *plc*-A, *plc*-B, and *plc*-C, are closely located, constituting an operon, whereas *plc*-D is located in a different region [8,9]. Moreover, polymorphisms frequently affect PLC genes in Mtb, as observed in different clinical isolates [10].

The importance of PLC in mycobacterium virulence was brought out by the demonstration that triple Δ*plcABC* and quadruple Δ*plcABCD* Mtb mutants attenuated tuberculosis infection in mice [6]. In addition, it has been previously shown that all Mtb PLCs present cytotoxic effects on macrophages *in vitro*. Recombinant PLC proteins expressed in *M. smegmatis* induced necrosis by hydrolysing membrane constitutive phospholipids into diacylglycerol (DAG) [7]. *C. perfringens-*PLC also induces cell necrosis through releases of DAG from host membrane by a mechanism dependent on activation of PKC, MEK/ERK, and NFkB pathways, leading to high concentrations of reactive oxygen species (ROS) and oxidative stress [11].

An increasing number of studies have highlighted the relationship between lipid mediators and cell death. Also, subversion of host eicosanoid biosynthetic pathways has been used as an evasion mechanism by a virulent mycobacterium [12]. It has been recently shown that infection with the attenuated Mtb strain H37Ra resulted in abundant production of the COX-2 product prostaglandin E_2_ (PGE_2_), and consequently in activation of membrane repair mechanism. On the other hand, the virulent strain H37Rv induces the production of lipoxin A_4_ (LXA_4_), which is an inhibitor of COX-2 expression and favours necrosis in infected cells [13-15]. Thus, the lipid mediators PGE_2_ and LXA_4_ appear to exert opposing effects on Mtb-induced cell death in macrophages. Another central lipid mediator in Mtb infection is leukotriene B_4_ (LTB_4_). We have previously shown that inhibition of leukotriene synthesis increased susceptibility to mycobacterial infection and pointed out alveolar macrophages as the main target for immunostimulatory actions of LTB_4_[16,17].

Given that mycobacterial PLCs have been associated with cell death, in this study we investigated whether this effect is related to the modulation of lipid mediator production induced by PLCs. Using two Mtb clinical isolates bearing genetic variations that affect PLC genes, we investigated how PLCs affect the outcome of Mtb-driven alveolar macrophage death and its relationship with lipid mediator production.

## Results

### PLCs-expressing *Mycobacterium tuberculosis* is more resistant to microbicidal activity and is associated with alveolar macrophage death

**Figure 1:**
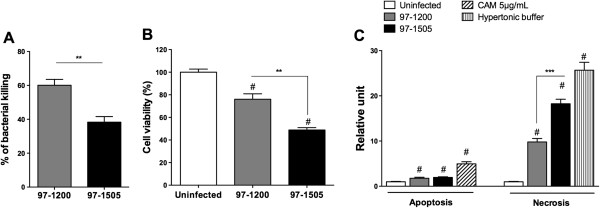
**Intracellular killing of Mtb isolates 97-1200 or 97-1505 and cell death of infected alveolar macrophages.** Alveolar macrophages were infected *in vitro* for 24 h with Mtb isolates 97-1200 or 97-1505 at MOI 5. **(A)** Bacterial killing was assessed by resazurin metabolisation and expressed as a percentage of phagocytised bacteria. **(B)** Cell viability assessed by resazurin metabolisation. Maximum viability (100%) is based on uninfected cells. **(C)** ELISA assay of apoptosis and necrosis 24 h post-infection of alveolar macrophages *in vitro*. Camptothecin 5 μg/mL (CAMP) was used as apoptosis-positive control and hypertonic buffer as necrosis-positive control. ^#^*P* < 0.0001 for uninfected cells vs. infected cells (97-1505 or 97-1200); ***P < 0.0001; **P < 0.001 (one-way ANOVA). Data are representative of three **(A, B)** and two **(C)** independent experiments (error bars, s.e.m.).

The virulence phenotypes of the isolates 97-1200 and 97-1505 were compared regarding the resistance or susceptibility to alveolar macrophage microbicidal activity. As shown in Figure 1A, after 24 hours of infection, the isolate 97-1505 (presence of PLCs) was more resistant to killing by alveolar macrophage than 97-1200 (absence of PLCs). Considering that mycobacterial PLCs have cytotoxic effects on macrophages [7], we studied the viability of rat alveolar macrophages infected *in vitro* with the isolates 97-1200 or 97-1505 to investigate if cell death is associated to mycobacterial PLCs. In comparison to uninfected cells, mycobacterium isolate 97-1505 reduced cell viability by more than 40%, which was approximately 20% higher than the cell death induced by 97-1200 (Figure 1B). Regarding the cell death modality, alveolar macrophages infected with 97-1505 underwent significantly more death by necrosis, and no differences were observed in apoptosis induced by 97-1200 or 97-1505 isolates (Figure 1C). These results suggest that Mtb bearing PLCs genes plays a role in host-cell death by inducing necrosis, which contributes significantly to mycobacterial resistance to microbicidal activity of alveolar macrophages.

### PLCs-expressing *Mycobacterium tuberculosis* more efficiently stimulates the production of proinflammatory cytokines and NO by alveolar macrophages *in vitro*

**Figure 2:**
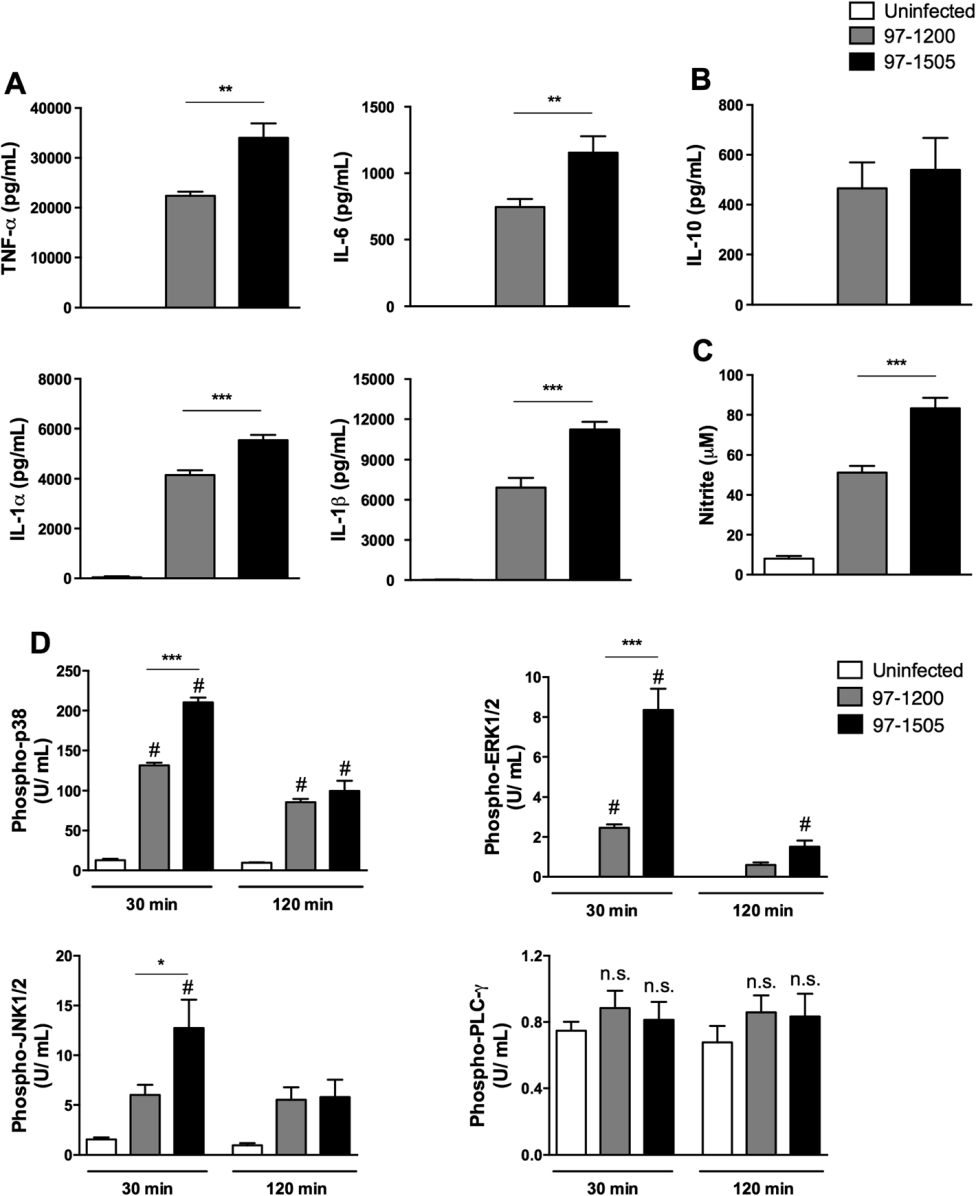
**PLC-expressing *Mycobacterium tuberculosis *more efficiently stimulates the cell activation, production of proinflammatory cytokines and NO**_**2 **_**in alveolar macrophages.** Production of **(A)** the proinflammatory cytokines TNF-α, IL-1α, IL-1β, and IL6; **(B)** IL-10, determined by ELISA, and **(C)** NO, determined by Greiss reaction. **(D)** Quantification of phosphorylated p38, ERK1/2, JNK1/2, and PLC-γ determined by CBA (Cytometric Bead Array), and expressed as U/ mL. # *P* < 0.0001 for uninfected cells vs. infected cells (97-1505 or 97-1200); ***P < 0.0001; *P < 0.05 (one-way ANOVA). Data are representative of three **(A–C)** and two **(D)** independent experiments (error bars, s.e.m.).

The results shown in Figure 1 indicate that the isolate 97-1505 is more resistant to bactericidal activity by inducing host-cell necrosis. Thus, we next asked if the production of pro-inflammatory cytokines and NO is affected, since these mediators are essential for host control of Mtb infection [18]. In addition, previous data from our lab revealed that lungs from mice infected with the isolate 97-1505 presented extended tissue damage, which was suggested to be associated with strong production of pro-inflammatory cytokines (data not shown). Here, *in vitro* infection showed that both isolates induced a strong production of NO and the cytokines TNF-α, IL-6, IL-1α, IL-1β, and IL-10. However, the amount of inflammatory cytokines and NO released in response to the 97-1505 isolate was significantly higher than that induced by the 97-1200 isolate (Figure 2A and C). Despite the increased production of IL-10, no difference was observed between macrophages infected with the two different isolates (Figure 2B).

We also evaluated the ability of PLCs to activate cell-signalling. Kinase proteins are directly associated to cytokine production in pro-inflammatory cell responses to bacterial stimulus [19], including Mtb [20]. Also, considering that other bacterial PLCs were previously reported to trigger host-cell signalling pathways [2,21], we sought to verify if the mycobacterial isolates from this study differentially activate cell-signalling proteins. Alveolar macrophages infected with both Mtb isolates showed increased phosphorylation of three serine-threonine protein kinases: MAPK p38, ERK1/2, and the c-Jun N-terminal kinase JNK1/2. Notably, the isolate 97-1505 induced higher levels of kinase phosphorylation than 97-1200 after 30 minutes of bacteria–host cell contact. On the other hand, host PLC-γ was not activated by either isolate (Figure 2D). These data suggest that PLC, as a mycobacterial virulence factor, plays a role in the cell activation and induction of proinflammatory cytokines by alveolar macrophages.

### PLCs-expressing *Mycobacterium tuberculosis* impaired COX-2 and PGE_2_/LTB_4_ receptor mRNA expression

**Figure 3:**
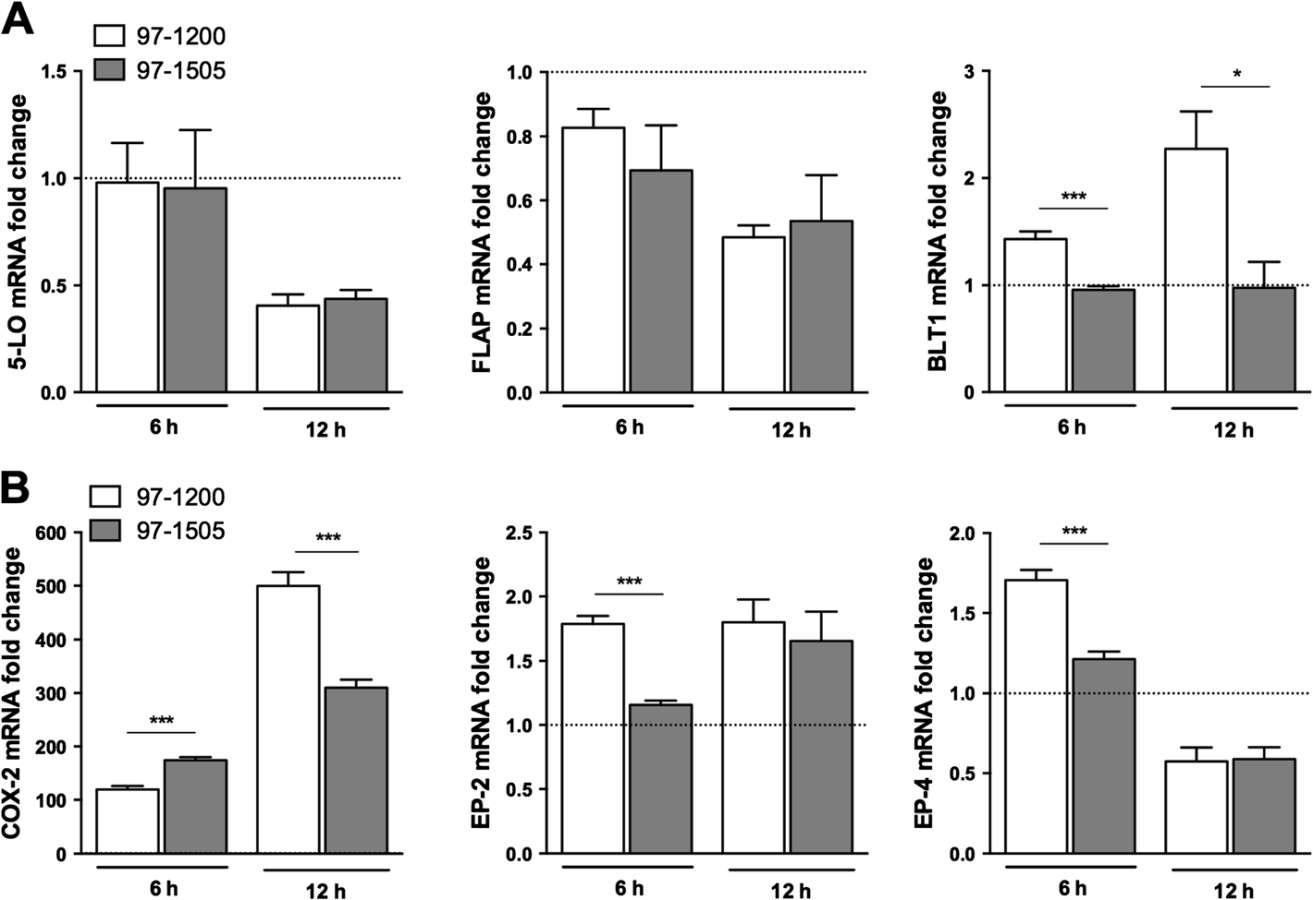
**Differential mRNA expression of COX-2 and PGE**_**2**_**/LTB**_**4 **_**receptors induced by Mtb isolates 97-1200 and 97-1505.** mRNA expression of **(A)** 5-LO, FLAP, and BLT1, and **(B)** COX-2, EP-2, and EP-4 in alveolar macrophages infected for 6 and 12 h with Mtb isolates 97-1200 and 97-1505. Dotted lines show the relative expression of uninfected cells (fold change = 1). All samples were normalised by *Gapdh* endongenous control. ***P < 0.0001; *P < 0.05 (one-way ANOVA). Data are representative of two independent experiments (error bars, s.e.m.).

Virulent Mtb uses the control of host-cell death pathways as a strategy to avoid immune response through subversion of host eicosanoid biosynthetic pathways [14]. Thus, to investigate if the PLCs represent a virulence advantage to the bacillus, we next evaluated the expression of mRNA for enzymes and receptors involved in the eicosanoid synthesis, such as 5-lipoxygenase (5-LO), 5-LO Activating Protein (FLAP), Leukotriene B_4_ (LTB_4_) receptor (BLT1), cyclooxygenase-2 (COX-2), and the PGE_2_ receptors EP-2 and EP-4. No differences were observed in 5-LO or FLAP mRNA expression induced by the Mtb isolates. On other hand, the isolate 97-1200 induced higher expression of BLT1 gene (*Ltb4r*), which is known to bind LTB_4_ and thus is related to antimicrobial defence (Figure 3C) [16,17,22]. Differential expression was also observed for genes related to the PGE_2_ synthesis pathway. During the first hours of Mtb infection (6 h), the expression of the inducible gene *Ptgs2*, which encodes COX-2, was higher in response to the 97-1505 isolate. After 12 hours, although the increased *Ptgs2* expression was maintained*,* it was lower than that induced by Mtb 97-1200. Associated with COX-2 induction, gene expression of the prostaglandin receptors EP-2 and EP-4 was also higher in alveolar macrophages infected with 97-1200, 6 hours after infection (Figure 3B). These findings suggest that PLCs-expressing *Mycobacterium tuberculosis* subverts the eicosanoid synthesis pathway by inhibiting COX-2, EP-2, and EP-4 expression, thereby directly influencing the generation of PGE_2_ and its related cellular response.

### Eicosanoid production is differentially induced by PLC-expressing *Mycobacterium tuberculosis* during alveolar macrophages infection

**Figure 4:**
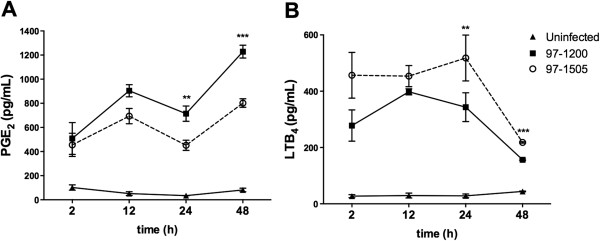
**LTB**_**4 **_**and PGE**_**2 **_**production by alveolar macrophages is differentially induced by PLC-expressing *Mycobacterium tuberculosis*.** Cells were infected with Mtb isolates 97-1200 or 97-1505 for 2, 12, 24, and 48 hours and the eicosanoid production was assessed in the supernatants by ELISA. ***P < 0.0001; **P < 0.001 (one-way ANOVA). Data are representative of three **(A)** and two **(B)** independent experiments (error bars, s.e.m.).

To study whether the modulation of COX-2 and eicosanoid receptor expression by the 97-1505 Mtb has effects on the biosynthesis of these mediators, we quantified PGE_2_ and LTB_4_ production by Mtb-infected alveolar macrophages at different time points. Figure 4A shows that 12 h after infection, PGE_2_ production induced by 97-1505 Mtb was similar to that induced by 97-1200 Mtb. However, after 24 h, 97-1505 Mtb-induced PGE_2_ production decreased drastically and remained lower at 48 h post-infection. Differently, 24 and 48 h after infection, LTB_4_ production induced by the isolate 97-1505 was higher than that induced by 97-1200 (Figure 4B). Together, our results support the idea that PLCs-expressing Mtb are involved in decreased PGE_2_ production and lower EP-2/4 gene expression, impairing eicosanoid-signalling pathway in alveolar macrophages.

### Cell death and subversion of PGE_2_ production are dependent on mycobacterial PLCs

**Figure 5:**
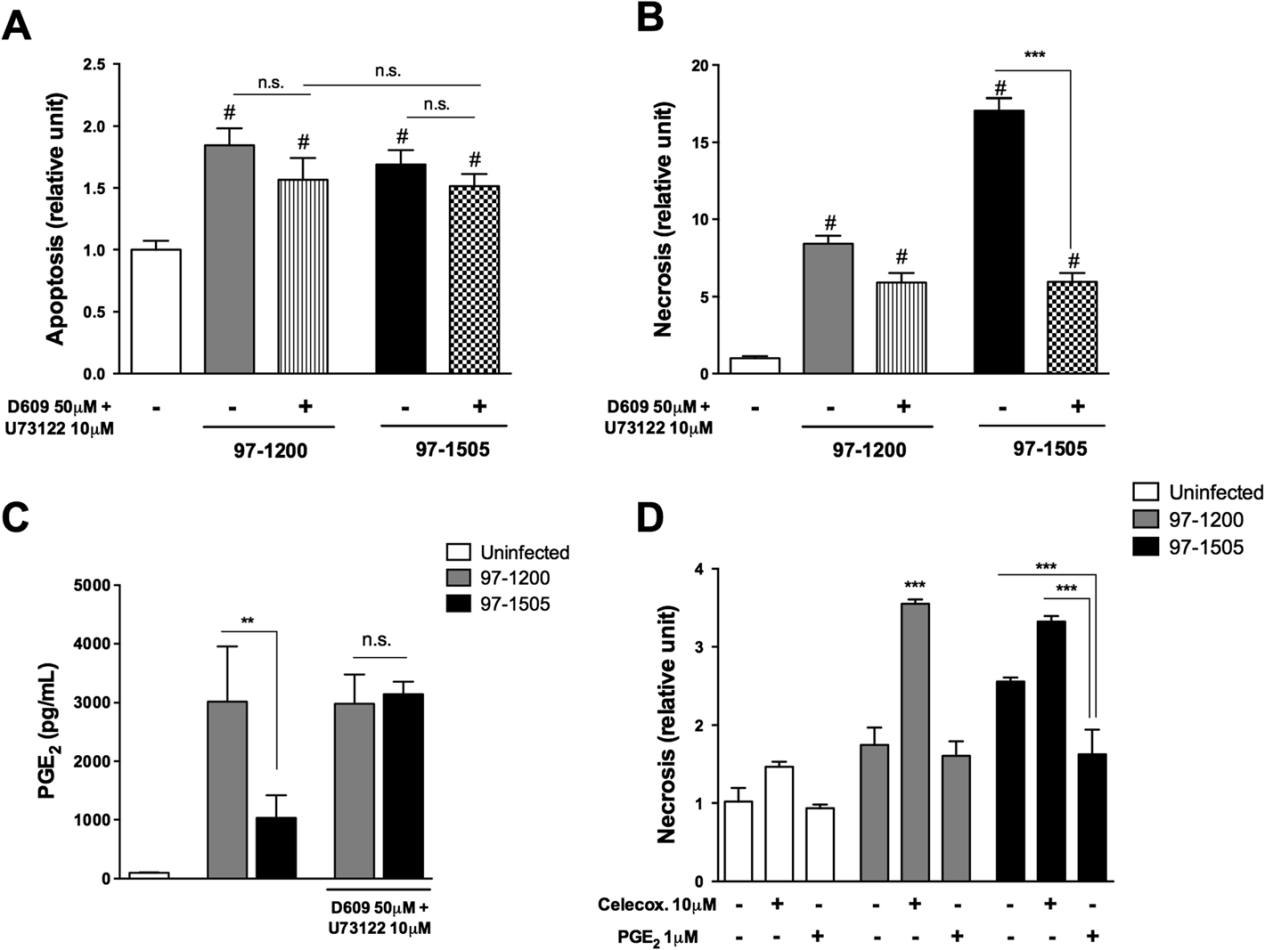
**PLC-expressing *Mycobacterium tuberculosis *induces alveolar macrophage necrosis through the regulation of PGE**_**2 **_**synthesis.** Alveolar macrophages were infected *in vitro* for 24 h with Mtb isolates 97-1200 or 97-1505 treated or not with the PLC inhibitors D609 (50 μM) and U73122 (10 μM). **(A, B)** ELISA assay of apoptosis and necrosis. **(C)** PGE_2_ production was assessed in supernatants by ELISA. **(D)** Celecoxib or PGE_2_ were added to the culture of alveolar macrophages infected or not with 97-1200 or 97-1505 and necrosis was assessed by ELISA. ^#^*P* < 0.0001 for uninfected cells vs. infected cells (97-1505 or 97-1200); ***P < 0.0001; **P < 0.001 (one-way ANOVA). Data are representative of three **(A, B)** and two **(C, D)** independent experiments (error bars, s.e.m.).

Thus far, our results showed that the Mtb isolate 97-1505 induces necrotic death in alveolar macrophages, which is associated with lower expression of COX-2 and PGE_2_ receptors, leading to reduced production of PGE_2_, compared with infection by 97-1200. We next examined whether these effects are PLC-dependent. To do that, we used the widely described PLC inhibitors D609 and U73122 [23-25]. Thus, we pre-incubated both Mtb isolates with PLC inhibitors (U73122 and D609) separately or combined (Additional file 1: Figure S1), and analysed the ability of the bacilli to cause necrosis and the effect on PGE_2_ production. The treatment of Mtb isolates with PLC inhibitors severely reduced necrosis of 97-1505-infected cells, whereas it did not affect the necrosis of PLC-deficient 97-1200-infected cells. Moreover, treatment with PLC inhibitors had no effect on apoptosis induced by both isolates (Figure 5A, B and Additional file 2: Figure S2A). Likewise, PGE_2_ production by Mtb 97-1505-infected alveolar macrophages presented levels similar to those produced by 97-1200-infected cells, and PLC inhibition did not affect the PGE_2_ production in cells infected by 97-1200 (Figure 5C and Additional file 2: Figure S2B). Finally, to address the role of PGE_2_ in cell death, celecoxib, a COX-2 inhibitor, was added to the culture, which increased necrosis rate in cells infected with both isolates. On the other hand, addition of PGE_2_ prevented cell necrosis during infection with the isolate 97-1505 (Figure 5D and Additional file 2: Figure S2C). Taken together, these data reinforce that infection with Mtb harbouring PLCs induces host-cell necrosis, which may be related to the subversion of PGE_2_ synthesis.

## Discussion

The central finding of this study was that PLC-expressing *Mycobacterium tuberculosis* is more virulent than Mtb lacking these enzymes, through inducing necrosis of alveolar macrophages, which is associated to subversion of PGE_2_ production. This is the first study to demonstrate such a role for mycobacterial PLCs using clinical isolates, which actually cause tuberculosis, instead of models of recombinant expression of these enzymes in non-pathogenic mycobacteria.

We showed that PLC-expressing Mtb (isolate 97-1505) induced high rates of alveolar macrophage death, especially through necrosis, whereas the PLC-deficient Mtb (isolate 97-1200), despite its ability to cause cell death, did not induce necrosis as efficiently. Control of macrophage cell-death pathways by Mtb has been extensively described as a strategy to avoid innate and adaptive immune responses [12,26,27]. Manipulation of cell-death modality has been successfully used by other intracellular pathogens such as *Chlamydia*, *Legionella pneumophila, Listeria monocytogenes, Shigella flexineri*, and *Salmonella enterica* subsp. *enterica* serovar Typhimurium [28-30]. It has been demonstrated that host-cell apoptosis confers protection to the host, once the uptake of apoptotic bodies derived from macrophages by dendritic cells allows an effective activation of the immune response [31]. In contrast, host-cell necrosis can benefit the pathogen because disruption of the cell membrane releases the bacteria to efficiently spread and infect adjacent cells [32]. Recently, descriptions of the manipulation of cell-death fate by Mtb have shown that a virulent bacillus, the H37Rv strain, caused macrophage necrosis whereas the attenuated strain H37Ra was related to apoptotic death [12]. Likewise, a Ndk- (nucleoside diphosphate kinase) knockout Mtb showed reduced virulence, which was demonstrated by the susceptibility to macrophage microbicidal activity and increased ability to induce host-cell apoptosis [33].

Pulmonary macrophages are the primary niches for Mtb replication, thus host resistance is critically dependent on innate immune functions played by these cells. In this scenario, proinflammatory cytokines and nitric oxide (NO) are essential for host control of Mtb. Macrophage recognition and phagocytosis of Mtb stimulates mostly the production of TNF-α, IL-1α and β, and IL-6, which are fundamental for the resolution of Mtb infection in mice [18]. Our results highlighted the proinflammatory response triggered by 97-1505 Mtb isolate, which induced a higher production of those cytokines by alveolar macrophages than the isolate 97-1200. Surprisingly, the higher production of proinflammatory cytokines did not result in better outcome for the host cell, as shown by the decreased macrophage survival. Stimulation of NO generation can cause oxidative stress leading to dysfunction in mitochondrial respiration and also block caspase-3 activity by nitrosylation, which may inhibit apoptosis and thereby promote necrosis [34]. Beyond the effects on the immune response, TNF-α has been associated with necrosis in a caspase-independent mechanism through activation of receptor TNFR1 and engagement of RIP1 kinase [34]. Recently, it was suggested that alveolar macrophages infected by an attenuated BCG (Bacillus Calmette–Guérin) show high expression of the TNF-α-receptor TNFR1 associated with increased cell apoptosis [35]. However, in that particular study, only apoptosis rate was analysed and necrosis was not shown. In addition, host-cell necrosis induced by the T3SS pore-forming protein, YopB, from pathogenic *Yersinia* has been associated with increased production of proinflammatory cytokines, such as IL-1β and TNF-α [36]. These findings support our data showing that proinflammatory cytokines are involved in cell death induced by intracellular bacteria.

Activation of the MAPK pathway has been directly linked to cytokines production in proinflammatory cell responses to bacterial stimulus [19], including Mtb [20]. In addition, MAP kinases have an essential role in production of lipid mediators, such as LTB_4_, since activation of 5-LO is dependent on phosphorylation mediated by ERK1/2 and p38 [37]. In this study, higher phosphorylation of MAPK p38, ERK1/2, and JNK1/2 was observed in cells infected with 97-1505. Although phosphorylation of ERK1/2 and p38 can also be triggered by mammalian PLCs, as demonstrated by LPS activation of the PLC–PKC pathway [38], we observed no differences in PLC-γ phosphorylation induced by the Mtb isolates 97-1200 or 97-1505 when compared to uninfected cells. Moreover, different mycobacterial PLC isoforms can trigger MAPK signalling by directly activating PKC through DAG production from cell membrane phospholipids [7,39]. Based on these findings, we hypothesise that the differential activation of the MAPK pathway in 97-1505-Mtb-infected alveolar macrophages may be due to mycobacterial PLC actions.

Macrophages infected by mycobacteria increase the production of LTB_4_ itself [17], which mediates host immunopathology by enhancing Th1 responses and by exacerbating inflammation [16,40]. LTB_4_ production induced by both isolates in this study was considerably amplified by PLCs; however, no significant differences were observed at the early stages of infection, which suggests that, besides PLCs, other mechanisms such as the overproduction of proinflammatory cytokines can contribute to immunopathology of Mtb infection. The emergent knowledge that the balance in LTB_4_ production is fundamental for the outcome of Mtb infection points out that the excessive production of this lipid mediator, associated to dysregulated production of TNF-α, increases Mtb susceptibility in the zebrafish model, demonstrated by necrosis of infected macrophages [41]. We also found a lower production of PGE_2_ to be associated with decreased mRNA expression of COX-2 and EP-2/4 receptors in Mtb 97-1505-infected alveolar macrophages. Our group previously demonstrated that pharmacological inhibition of COX-2 results in increase of LTB_4_ synthesis, during Mtb infection in mice [17]. In the present study, we show that addition of exogenous LTB_4_ to the culture impairs PGE_2_ production by infected cells. These data are in accordance with the concept of a shift in lipid mediator production toward one eicosanoid subpathway [42], which may explain the higher LTB_4_ and lower PGE_2_ production observed here. Moreover, the finding that down-regulation of PGE_2_ and higher necrosis were both impaired after incubation of the isolate 97-1505 with PLC inhibitors, supports the hypothesis that virulent mycobacterium subverts eicosanoid synthesis to manipulate host-cell death to promote proliferation and dissemination [15]. Here, when exogenous PGE_2_ was added to 97-1505-infected alveolar macrophages, the necrosis rate decreased. On the other hand inhibition of PGE_2_ by celecoxib enhanced necrosis in cells infected by both isolates. It has been reported that PGE_2_-preventing necrosis is due to PGE_2_ involvement in the synthesis of the lysossomal Ca^2+^ sensor SYT7, which is essential for prevention of mitochondrial damage, enabling repair of plasma membrane disruption [14]. Although virulent mycobacteria sabotage of PGE_2_ to induce necrosis has been associated with increased production of LXA_4_[12,13,41], we did not detect LXA_4_ in the supernatant of Mtb-infected alveolar macrophages (data not shown). Nevertheless, the potential relationship between mycobacterial PLCs and host-cell necrosis through down-regulation of PGE_2_ production shown in this study is new evidence of the relevance of this virulence factor.

Indeed, despite the described *plc* gene polymorphism [10], there is no genome or proteome characterised for either Mtb isolate, and further studies are necessary to better understand the differences between 97-1505 and 97-1200, and the role of PLC in Mtb virulence. However, our data make a valuable demonstration of subversion of lipid mediator synthesis and its association with cell necrosis. Furthermore, our data are consistent with the recent finding of Bakala N’Goma and colleagues [7], who showed for the first time the cytotoxic effect of mycobacterial PLCs on macrophages. Finally, the relevance of PLCs as determinants of virulence in Mtb expands our understanding of how these virulence factors can act to the detriment of the host, and highlights eicosanoids, such as PGE_2_ and LTB_4_, as mediators with functions that extend beyond innate immune mechanisms.

## Conclusion

We found that the *Mycobacterium tuberculosis* bearing PLCs genes is more resistant to microbicidal activity of alveolar macrophages and induces cell necrosis, which is associated with subversion of PGE_2_ production.

## Methods

### *Mycobacterium tuberculosis* isolates

The clinical isolates 97-1505 and 97-1200 were obtained from patients with active tuberculosis in 1998 and belong to a collection of 790 strains from RIVM (Bilthoven, The Netherlands). Both isolates were characterised regarding the polymorphisms in *plc* genes. The former has the entire *plc*-A and *plc*-B genes and an insertion of a copy of IS*6110* at *plc*-C and the latter has all *plc* genes deleted. Also, analysis of the RFLP (Restriction fragment length polymorphism) pattern revealed similarities greater than 70% in the IS*6110*-RFLP profiles between the isolates [10]. Cultures were grown on Lowenstein-Jensen (LJ) solid medium then transferred to Middlebrook 7H9 (Difco, Detroit, MI) liquid medium supplemented with OADC (Difco). The culture was harvested by centrifugation, and the cell pellet was resuspended in sterile phosphate-buffered saline (PBS) and the number of bacteria was adjusted to 1 × 10^7^ bacteria/ mL by absorbance in DO_600nm_. Viability of Mtb suspension was tested previously with fluorescein diacetate and ethidium bromide (Sigma, St. Louis, MO) [43] and by resazurin metabolisation within 24 h (Additional file 3: Figure S3A). In some experiments, the Mtb isolates were pretreated with 10 μM of U73122 (phosphatidylinositol-phospholipase C inhibitor (PI-PLC) – Calbiochem, San Diego, CA) and with 50 μM of D609 (phosphatidylcholine-specific phospholipase C inhibitor (PC-PLC) – Calbiochem, San Diego, CA) for 1 h at 37°C with agitation. To test the efficiency of these inhibitors, recombinant PLC from *Clostridium perfringens* was used and the PLC activity was assessed by the p-NPPC assay [44] (Additional file 4: Figure S4). After that, all suspensions were centrifuged at 3,500 rpm for 10 min and washed twice with PRMI before addition to alveolar macrophage cultures. All experiments using mycobacterium isolates were conducted in a biosafety level 3 laboratory (BSL-3), according to permission of Brazilian national authorities (registration number 003097).

### Cell isolation, culture, and *in vitro* infection of alveolar macrophages

Resident rat alveolar macrophages of > 95% purity were obtained from *ex vivo* lung lavage [45] and resuspended in RPMI 1640 at 2 × 10^6^ cells/ml. Cells were adhered to tissue culture-treated plates for 2 h (37°C, 5% CO_2_) and were cultured overnight in RPMI containing 10% FBS and 1% gentamicin. Before performing the experiments, cells were washed two times with warm medium to remove nonadherent cells. Cells were infected with Mtb isolates 98-1200 and 97-1505 at MOI 5 and incubated for 2 h, followed by two washes and a further incubation of cells in fresh medium for another 4, 10, 22, or 46 hours, depending on the experiment. In some experiments, celecoxib (10 μM), PGE_2_ (1 μM), or LTB_4_ (1 μM) were added to the cultures during Mtb infection. All experiments were approved and conducted in accordance with guidelines of the Animal Care Committee of Universidade de São Paulo (Protocol nº 11.1.252.53.3).

### Measurement of eicosanoids, cytokines and NO

PGE_2_ and LTB_4_ concentrations in cell supernatants were determined using ELISA EIA kits (Cayman Chemical, Ann Arbor, MI). Cytokine concentrations were determined using a Duoset ELISA Development kit (R&D Systems, Minneapolis, MN), according to the manufacturer’s recommendations. NO production was assessed by detection of nitrite concentration in cell supernatants using the Greiss reagent (0.1% NEED and 1% sulfanilamide). Values were determined using a standart curve based in serial dilutions of NaNO_2_.

### Resazurin assay of cell viability and bacterial killing

The resazurin assay has been used as a rapid test for evaluating mammalian cell or microorganism viability and as a cytotoxic susceptibility assay, in which the system incorporates an oxidation-reduction (REDOX) indicator, generating a fluorescent metabolite [46]. Alveolar macrophages were plated in 96-well dishes at 2 × 10^5^ cells/well. After infection time, 10 μL of a resazurin solution (0.5 mg/mL) (Sigma, St. Louis, MO) was added to each well and cells were incubated for 8 hours for viability evaluation (37°C, 5% CO_2_). Fluorescence level was measured by a fluorescent microplate reader (SpectraMax Paradigm, Molecular Devices, Sunnyvale, CA) with excitation at 560 nm and emission at 590 nm. To assess the bacterial killing, the Mtb isolates were added at MOI 5 to alveolar macrophage cultures in two 96-well plates. After 2 h of incubation, the supernatant was removed and the cells washed three times with PBS to remove non-phagocytised bacteria. In one of the plates, cells were replenished with fresh medium and incubated for a further 22 h. In the other plate, alveolar macrophages were lysed using 200 μL of 0.05% saponin, then 10 μL of a resazurin solution was added to each well and phagocytised bacteria in suspension were incubated (37°C, 5% CO_2_) for 24 hours for further assessment of fluorescence level (Additional file 3: Figure S3B). The remaining plate, after 24 h of incubation, was submitted to the same wash and resazurin procedure. Bacterial killing was expressed as the percentage relative to phagocytised bacteria.

### *In vitro* necrosis and apoptosis assays

Evaluation of apoptosis and necrosis in alveolar macrophages was performed as previously described [14] by ELISA assay cell (Cell Death Detection ELISA^PLUS^; 11 774425 001; Roche Applied Science, Mannheim, Germany), which allows the quantification of cytoplasmic (apoptosis) and extracellular (necrosis) histone-associated DNA fragments. The relative amount of necrosis or apoptosis was calculated as a ratio of the absorbance of infected macrophages to that of uninfected control macrophages. Camptothecin (Sigma, St. Louis, MO) 5 μg/mL was used as apoptosis-positive control and a hypertonic buffer (10 mM Tris, pH 7.4; 400 mM NaCl; 5 mM CaCl_2_ and 10 mM MgCl_2_) as necrosis-positive control.

### Analysis of gene expression by real-time polymerase chain reaction (PCR)

Total RNA was extracted from 4 × 10^6^ alveolar macrophages using Trizol® reagent (Invitrogen) according to the manufacturer’s instructions, and cDNA synthesis was performed using the cDNA High Capacity Archive kit (Applied Biosystems, Foster City, CA). Subsequently, the mRNA expression was evaluated by real-time PCR using the TaqMan® method. Briefly, the reaction mixture contained 12.5 ng of cDNA, 5 μL of TaqMan® Universal PCR Master Mix, and 0.5 μL of TaqMan specific primer/probe (Applied Biosystems) in a 10 μL final volume reaction. For each experiment, samples (n = 5-2) were run in duplicate. The probes used for amplification were synthesised using the Assay-on-Demand System (Applied Biosystems) with the following GeneBank sequences: *Ptgs*2 (NM_017232.3), *Ptger*2 (NM_031088.1), *Ptger*4 (NM_032076.3), *Alox*5 (NM_012822.1), *Alox*5ap (NM_017260.2) and *Ltb*4r (NM_021656.1). The 2^–ΔΔCT^ method was used in the analysis of the PCR data. First, the difference in gene expression was assessed between each gene and an endogenous control (*Gapdh*) for each sample to generate the ΔΔCT. The relative gene expression in each sample was determined as follows: relative amount of target = 2^–ΔΔCT^ value*.* Uninfected alveolar macrophages were used as control samples and their average values were set as 1. The relative gene expression for each experimental sample was compared with this value*.*

### Phosphoprotein detection by Cytometric Bead Array Flex Set

Samples were prepared according to the manufacturer’s protocol for adherent cells (Becton Dickinson, Heidelberg, Germany). Alveolar macrophages were stimulated by Mtb isolates 97-1200 or 97-1505 for 30 minutes, 1 hour, and 2 hours. Addition of denaturation buffer halted activation of cells and samples were placed immediately in a boiling water bath for 5 min. Cell lysates were centrifuged at 14,000 rpm for 5 min and supernatants were stored at –80°C until measurement of kinase phosphorylarion. Quantitative determination of pJNK1/2 (T183/Y185), pp38 (T180/Y182), pERK1/2 (T202/Y204), and pPLC-γ (Y783) was performed using antibodies from the multiplex Flex Set Cytometric Bead Array (Becton Dickinson, CA, USA). Afterwards, mixed capture beads and PE detection reagent were added to allow detection of phosphoprotein-antibody complexes. Flow cytometric analysis was performed using FACSCanto TM and a FACSDiva was used for data acquisition and analysis (Becton Dickinson, CA, USA). A total of 900 events were acquired.

### Statistical analysis

Data were evaluated by analysis of the variance (ANOVA) between groups followed by the Turkey’s correction post-test. In all comparisons, a significance level of *P* < 0.05 was considered to be significant.

## Competing interests

The authors declare that they have no competing interests.

## Authors' contributions

PAA: Conceived and designed the experiments; PAA, MSE, WMR, and PATP: Performed the experiments; PAA, MSE, and FWGPS: Analysed the data; LHF, SCL, and CLS: Contributed reagents/materials/analysis tools; PAA, MSE, FWGPS, and LHF: Wrote the manuscript. All authors read and approved the final manuscript.

## Supplementary Material

Additional file 1: Figure S1 Viabilidty of Mtb isolates after treatment with the PLC inhibitors D609 and U73122. Click here for file

Additional file 2: Figure S2 Inhibition of Mycobacterial PLCs affects alveolar macrophage necrosis through the regulation of PGE_2_ synthesis. Click here for file

Additional file 3: Figure S3 Resazurin metabolisation by Mtb isolates 97-1200 and 97-1505 and phagocytosis rate by alveolar macrophages. Click here for file

Additional file 4: Figure S4 PLC activity assay. Click here for file
