# Supplementary material for: Mycobacterium tuberculosis expressing phospholipase C subverts PGE2 synthesis and induces necrosis in alveolar macrophages
Source: BMC Microbiol. 2014 May 19;14:128. doi: 10.1186/1471-2180-14-128 (PMC4057917; doi:10.1186/1471-2180-14-128)
Supplement: Additional file 1: Figure S1 — Viabilidty of Mtb isolates after treatment with the PLC inhibitors D609 and U73122. [file 1471-2180-14-128-S1.pdf]

Figure S1

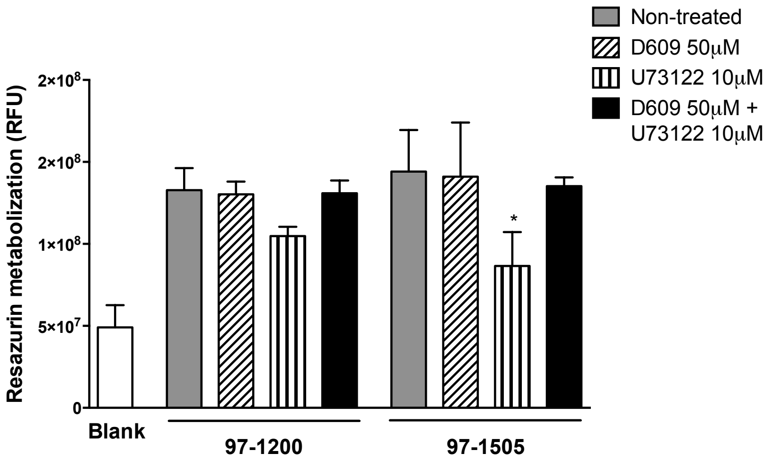

Figure S1- Viabilidty of Mtb isolates after treatment with the PLC inhibitors D609 and U73122 Bacterial resazurin metabolization was analyzed after incubation for 1h at 37°C with the PC-PLC inhibitor D609 (50 µM), or U73122 (10 µM) or either the combination of both. Cultures were then washed three times, resuspended in RPMI media and incubated with resazurin for 24 hours. **P* < 0.05 (one-way ANOVA). Data are representative of two independent experiments (error bars, s.e.m.).
