# Supplementary material for: Mycobacterium tuberculosis expressing phospholipase C subverts PGE2 synthesis and induces necrosis in alveolar macrophages
Source: BMC Microbiol. 2014 May 19;14:128. doi: 10.1186/1471-2180-14-128 (PMC4057917; doi:10.1186/1471-2180-14-128)
Supplement: Additional file 2: Figure S2 — Inhibition of Mycobacterial PLCs affects alveolar macrophage necrosis through the regulation of PGE2 synthesis. [file 1471-2180-14-128-S2.pdf]

Figure S2

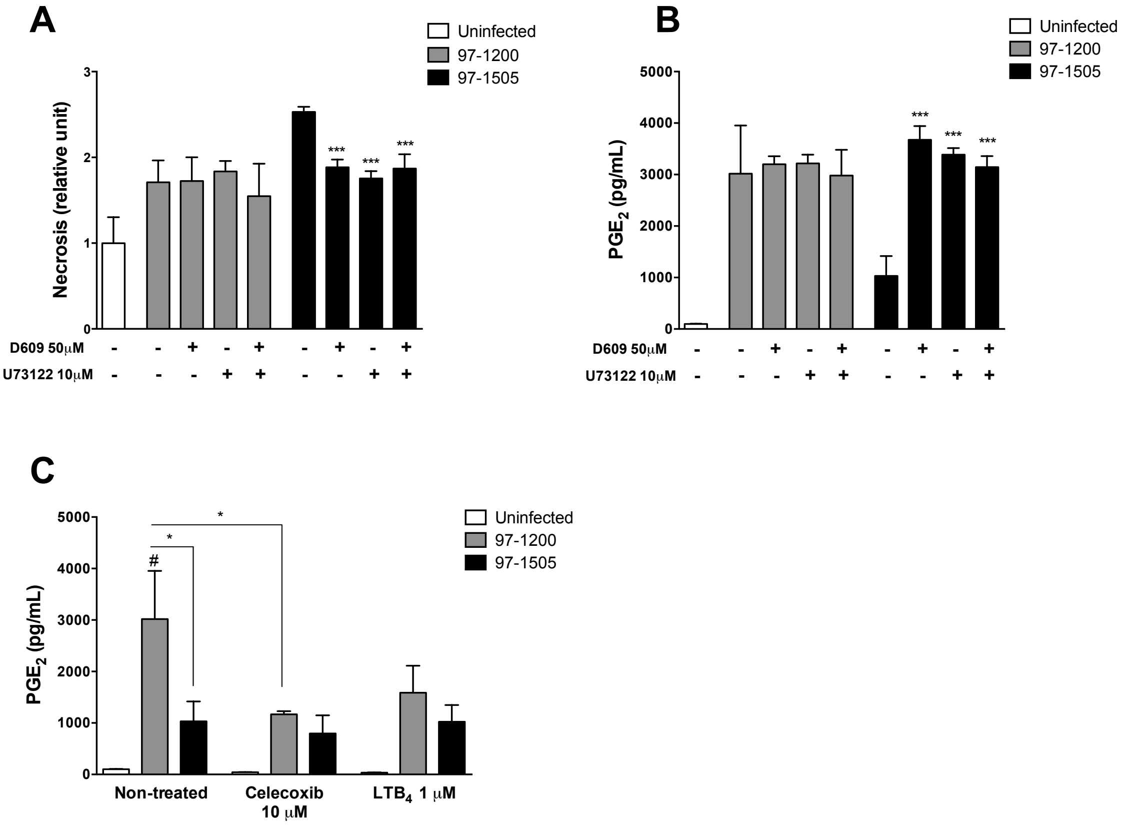

Figure S2- Inhibition of Mycobacterial PLCs affects alveolar macrophage necrosis through the regulation of PGE_2 synthesis. (A) Alveolar macrophages were infected *in vitro* for 24 h with Mtb isolates 97-1200 or 97-1505 treated or not with the PLC inhibitors U73122 (10 μM) and D609 (50 μM) separately or combined and necrosis rate was assessed by ELISA assay. (B) PGE_2 production was assessed in supernatants by ELISA. (C) Celecoxib and LTB_4 were added to the culture of alveolar macrophages infected with the isolates 97-1200 or 97-1505 to assess PGE_2 production by ELISA. $^{\#}P <$ 0.0001 for Uninfected cells vs. infected cells (97-1505 or 97-1200), $^{***}P < 0.0001$; $^{*}P <$ 0.05 (one-way ANOVA). Data are representative of two independent experiments (error bars, s.e.m.).
