# Supplementary material for: Mycobacterium tuberculosis expressing phospholipase C subverts PGE2 synthesis and induces necrosis in alveolar macrophages
Source: BMC Microbiol. 2014 May 19;14:128. doi: 10.1186/1471-2180-14-128 (PMC4057917; doi:10.1186/1471-2180-14-128)
Supplement: Additional file 3: Figure S3 — Resazurin metabolisation by Mtb isolates 97-1200 and 97-1505 and phagocytosis rate by alveolar macrophages. [file 1471-2180-14-128-S3.pdf]

Figure S3

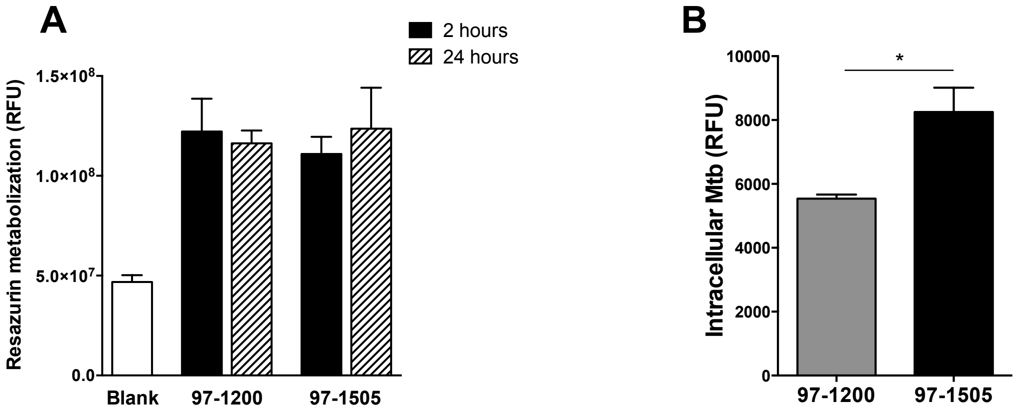

Figure S3- Resazurin metabolisation by Mtb isolates 97-1200 and 97-1505 and phagocytosis rate by alveolar macrophages. (A) Bacterial-resazurin metabolization was analyzed after incubation in RPMI for 2 hours (phagocytosis time) + 24h after addition of resazurin and 24h (killing time) + 24h after addition of resazurin. Blank shows RPMI + resazurin. (B) Alveolar macrophages were infected *in vitro* for 2 h with Mtb isolates 97-1200 or 97-1505 at MOI 5. Bacterial phagocytosis was assessed by resazurin metabolization of intracellular bacteria. RFU: Relative fluorescence unit. *$P < 0.05$ (*t* Test). Data are representative of four independent experiments (error bars, s.e.m.).
