# Supplementary material for: Mycobacterium tuberculosis expressing phospholipase C subverts PGE2 synthesis and induces necrosis in alveolar macrophages
Source: BMC Microbiol. 2014 May 19;14:128. doi: 10.1186/1471-2180-14-128 (PMC4057917; doi:10.1186/1471-2180-14-128)
Supplement: Additional file 4: Figure S4 — PLC activity assay. [file 1471-2180-14-128-S4.pdf]

Figure S4

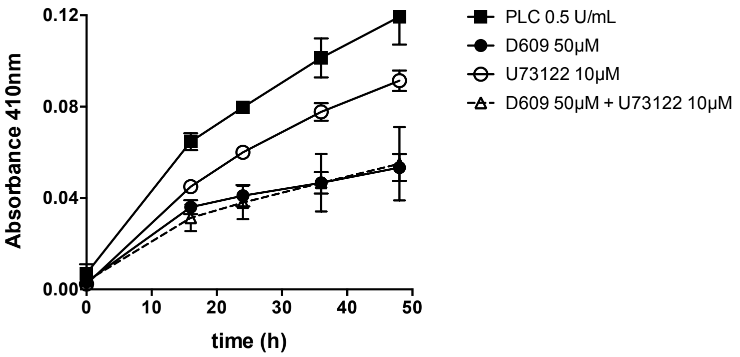

Figure S4- PLC activity assay. p-NPPC was incubated with 0.5 U/ml of PC-PLC from *Clostridium perfringens.* PLC inhibitors D609, U73122 or the combination of both were incubated 1 hour at 37 °C with the enzyme priors the addition of the substrate p-NPPC. Absorbance at 410 nm was determined after 0, 16, 24, 36 and 48 h. Data are representative of two independent experiments (error bars, s.e.m.).
